# Comparison of the Friction and Wear Characteristics between Copper and Paper Based Friction Materials

**DOI:** 10.3390/ma12182988

**Published:** 2019-09-15

**Authors:** Liang Yu, Biao Ma, Man Chen, Heyan Li, Chengnan Ma, Jikai Liu

**Affiliations:** 1 School of Mechanical Engineering, Beijing Institute of Technology, Beijing 100081, China; bright_yu@bit.edu.cn (L.Y.); mabiao@bit.edu.cn (B.M.); 2 College of Urban Transportation and Logistics, Shenzhen Technology University, Shenzhen 518118, China; liheyan@sztu.edu.cn; 3 Shanghai Automotive Industry Corporation Motor Technical Center, Shanghai 201804, China; machengnan@saicmotor.com; 4 China Institute of Microelectronics of Chinese Academy of Sciences, Beijing 100029, China; Liujikai@ime.ac.cn

**Keywords:** friction material, pin-on-disc test, coefficient of friction, wear depth

## Abstract

Copper-based friction material (CFM) and paper-based friction material (PFM) are the two most commonly used clutch friction materials. The friction and wear characteristics of these two kinds of friction materials under dry conditions were investigated by the pin-on-disc test over a broad range of applied loads, rotating speeds and ambient temperatures. Before experiments, the running-in test was conducted to stabilize the coefficient of friction (COF) and wear amount of the test samples. After experiments, the metallographic micrographs of the tested samples were presented to investigate the wear mechanisms. Experimental results showed that both the COF and wear depth of the CFM are much greater than these of PFM. The COF of CFM decreases with the increase of applied load, and changes slightly with the variation of rotating speed, whereas it increases first and then decreases with the increase of ambient temperature. However, the COF of PFM decreases dramatically with the increase of the rotating speed and ambient temperature, while it remains stable at first and then decreases slowly as the applied load increases. Additionally, under such three working conditions, the wear depth of CFM changes linearly, while the wear depth of PFM varies greatly. This study can be used as a guide for selecting friction materials for clutches with different applications.

## 1. Introduction

Friction materials play a significant role in the clutch system [1,2]. The friction properties of friction materials have a direct influence on the thermodynamic performance of the clutch, thus determining the reliability and safety of the entire vehicle [3]. In general, the selected friction materials should not only maintain a high and stable coefficient of friction (COF) to obtain better thermodynamic performance, but also have a low wear rate to extend the service life. Copper-based friction material (CFM) and paper-based friction material (PFM) are the two most commonly used friction materials in clutches.

The CFM manufactured by powder metallurgy has been widely applied to heavily-loaded wet clutches because of their excellent mechanical, thermal and tribological properties. A simplified experiment was developed to predict the friction performance of CFM wet clutches by the pin-on-disc test [4]. Zhao et al. [5,6] studied the friction and wear characteristics of the CFM by pin-on-disc tests; it was implied that temperature had the greatest influence on the wear loss of lubricated CFM clutches, and the COF witnessed a decrease after an increase with the increase of temperature. Su et al. [7,8] investigated the tribological properties of CFM with exogenous copper powder third body. Additionally, Kovalchenko et al. [9] studied the influence of the molybdenum disulfide and molybdenum diselenite on the friction and wear mechanisms of CFM under the dry condition. Gong et al. [10] not only established the wear map for CFM clutches under oil lubrication condition, but also developed linear equations to mark the transition boundaries between different wear regimes. Gyimah et al. [11] studied the effect of sintering temperature on the mechanical and tribological characteristics of the train brake pads; they found that high sintering temperature is helpful to increase the COF and reduce the wear amount. 

PFM is basically composed of fibrous reinforcements, binders, friction modifiers and fillers, etc. The fibers play a vital role in maintaining strength, thermal stability, and the friction performance of PFM. At present, various kinds of fibers—for example, carbon fiber, Kevlar fiber, ceramic fiber, and cellulose fiber—are gradually emerging to act as reinforcements in PFM [12,13,14,15]. The binder is the matrix of friction material, which determines the friction properties of materials [16]. The commonly used binders are phenolic resin and modified phenolic resin, which have good wear resistance and thermal stability [17,18]. Fei et al. [19] studied the influence of the carbon fiber content on the friction and wear performance of PFM. Rezaei et al. [20] investigated the effect of fiber length on the thermomechanical properties of short carbon fiber reinforced polypropylene composites. Yu et al. [21] studied the friction torque variations of PFM clutches during the engagement process, indicating that the operating factors had a dramatic influence on the stability of PFM. Additionally, Lu et al. [22] suggested that adding a small amount of organosilicon to PFM could increase the static COF while reducing the dynamic COF and wear rate. Li et al. [23] found that the wear mechanism of PFM was related to the thermal degradation and mechanical effects, and then they developed a methodology for predicting the wear of PFM.

It is reported that the main failure modes appearing in clutches are friction material degradation and the deformation of separate plate [24,25]. As the friction material degradation progresses, the surface topography changes gradually resulting in an increasing real contact area, meanwhile the mechanical and physical properties also change progressively. Considerable research efforts have been devoted to develop new friction materials, or to investigate the effects of additives on the overall friction properties of materials. However, little attention has been devoted to the evolution of friction and wear properties of friction material under various working conditions; there is no industry standard to judge the failure of friction materials. Moreover, researchers rarely compare the friction and wear characteristics of different friction materials with the same applications. Therefore, this study is performed to remedy these deficiencies.

Herein, the friction and wear characteristics of the CFM and PFM are investigated and compared experimentally. The influence of the working conditions (applied load, rotating speed and ambient temperature) on the variations of COFs and wear depths of friction materials is presented. The wear mechanisms of CFM and PFM are also explored. Finally, the conclusions provide guidance for selecting suitable friction materials for clutches with different applications.

## 2. Experimental Procedure

### 2.1. Experimental Equipment

**Figure 1:**
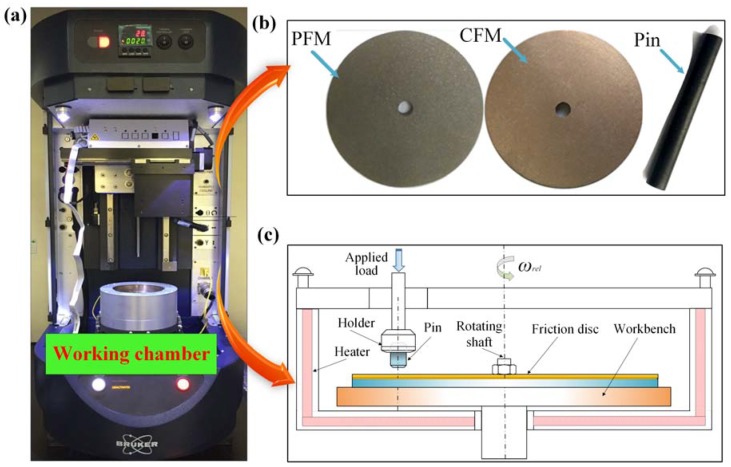
Experimental equipment and test samples (**a**) Universal Material Tester (UMT-5); (**b**) test samples; (**c**) simplified model of the working chamber.

The experimental equipment, Universal Material Tester (UMT-5) (Suzhou Tophung Company, Suzhou, China) simplified model of the working chamber in UMT-5 is presented in Figure 1c, where the pin is fixed by the holder and the friction disc rotates with the working bench. The sliding track of the pin is 25 mm away from the center of friction disc. The ambient temperature remains the pre-defined value by the heater, and the heating range is 0–1000 °C. As listed in Table 1, the experiments are divided into three working conditions with the applied load, rotating speed and ambient temperature taken into account. 

### 2.2. Friction Materials

The test samples are shown in Figure 1b, which were made in the Hangzhou PM Research Institute. To be more specific, the friction disc is composed of the friction core and friction material. Both the friction core and the pin are made of 65 Mn steel, while the friction material bonded to a friction core is made of CFM or PFM. The length and diameter of the pin are 50 mm and 6 mm, respectively. The thicknesses of friction core and friction material are 4 mm and 2 mm, respectively; the inner and outer diameters of the friction disc are 4 mm and 60 mm, respectively. CFM mainly contains Cu (70 wt%), Sn (6 wt%), Zn (5 wt%), SiO_2_ (5 wt%), graphite (6 wt%) and some other additive materials; while PFM mainly includes cellulose fiber (50 wt%), nitrile modified phenolic resin (25 wt%), BaSO_4_ (10 wt%) and kaolin (10 wt%), and some other additive materials. The thermophysical properties of the pin and disc are listed in Table 2.

### 2.3. Data Processing

**Figure 2:**
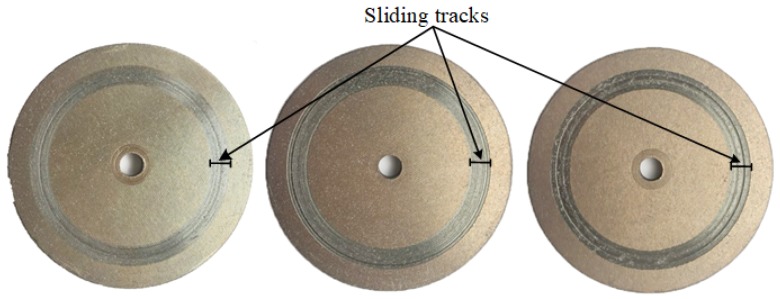
Discs after experiments.

As shown in Figure 2, deep scratches appeared on the sliding tracks after experiments, which directly reflected the wear status of a disc. Additionally, the depth of these scratches—own as the wear depth—can be measured by the position sensor in Z direction. Thus, the wear depth is used to evaluate the wear difference of CFM and PFM under different test conditions. Such a method can not only qualitatively reflect the wear amount of the disc, but also greatly reduce the complexity of the measurement of wear amount. Since the elastic modulus and hardness of 65 Mn steel are far higher than these of CFM and PFM, it is assumed that the wear mainly occurs on the disc. 

**Figure 3:**
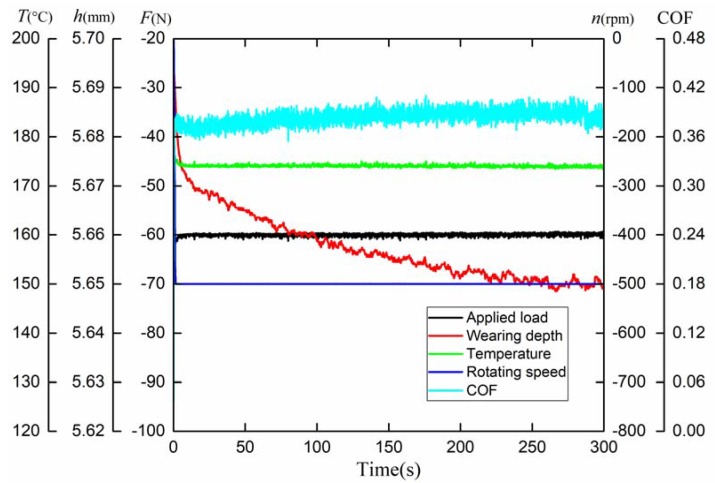
Representative test signals.

The representative test signals are demonstrated in Figure 3, including the applied load, rotating speed, ambient temperature, COF and wear depth. In the setting of the pressure sensor, it is stipulated that the tensile stress is positive and the compressive stress is negative; as for the speed sensor, the clockwise rotation is positive and the counterclockwise rotation is negative. In addition, the mean COF is introduced to evaluate the global friction properties of CFM and PFM, which can be calculated as follows.

(1) $$\bar{\mu }=\frac{\sum_{i=1}^{N}\mu_{i}}{N}$$

where $\mu_{i}$ and $\bar{\mu }$ are the instantaneous COF and mean COF, respectively. *N* is the total number of samples of the instantaneous COF.

## 3. Running-in Process

After the running-in experiments, the friction material will have better bearing characteristics, more stable COF, lower wear rate and energy loss, so as to obtain longer service life. Since the specific procedures and methods for running-in test have been described in our previous studies [26], only the results are presented in the study.

**Figure 4:**
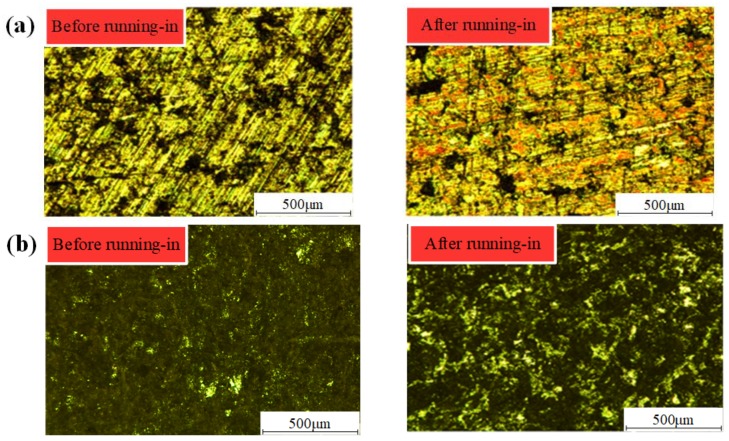
Metallographic micrograph of the disc before and after the running-in process (**a**) copper-based friction material (CFM); (**b**) paper-based friction material (PFM).

Figure 4 illustrates the metallographic micrographs of the disc before and after the running-in process. As shown in Figure 4a, during the running-in process, the tops of asperities on the CFM have undergone the plastic deformation and peel off gradually under the combined action of normal load and shear stress. Consequently, most of the asperities have been smoothed out, and the number of asperities gradually decreases, contributing to the stability of the friction performance. As shown in Figure 4b, the metallographic micrographs of PFM before and after the running-in test are quite different. Before the running-in test, the friction surface of PFM is mainly covered with fibers, which contributes to larger roughness; additionally, the COF is high due to the large shear stress generated by the asperity contact. After the running-in test, fibers are polished and a small amount of resin appears on the friction surface; the shear stress produced by asperities contact decreases and becomes stable gradually, the COF of PFM also keeps stable gradually.

## 4. Results and Discussion

**Figure 5:**
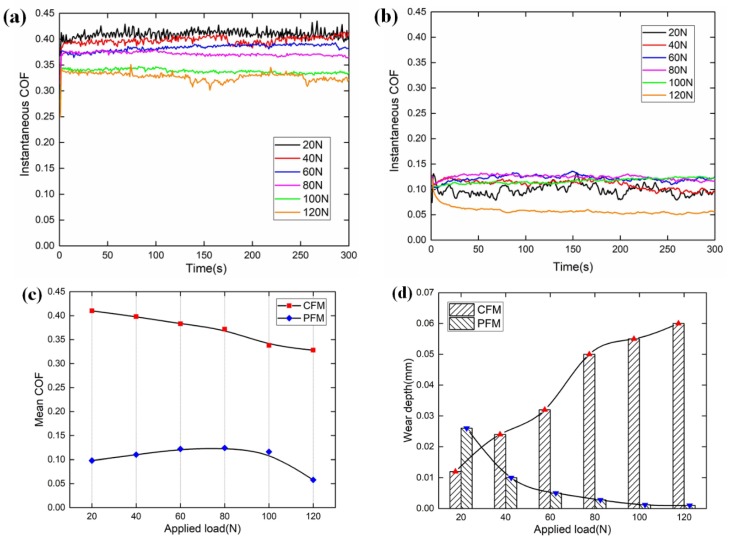
Experimental results under the applied load condition. (**a**) Instantaneous COF of CFM; (**b**) instantaneous COF of PFM; (**c**) mean COFs of the CFM and PFM; (**d**) wear depths of the CFM and PFM.

**Figure 6:**
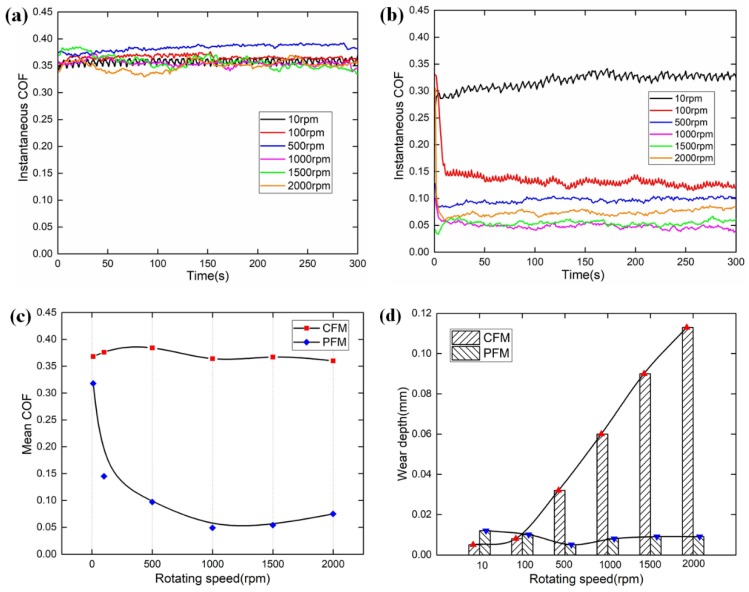
Experimental results under the rotating speed condition. (**a**) Instantaneous COF of CFM; (**b**) instantaneous COF of PFM; (**c**) mean COFs of the CFM and PFM; (**d**) wear depths of the CFM and PFM.

**Figure 7:**
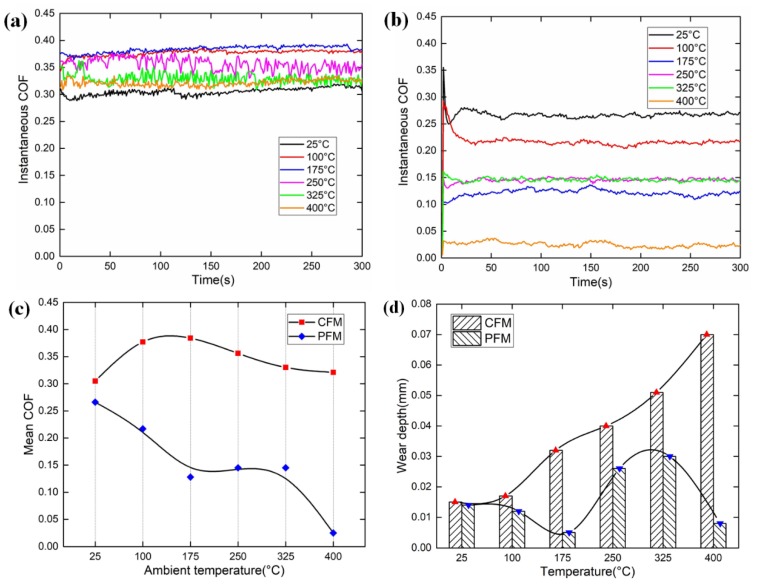
Experimental results under the ambient temperature condition. (**a**) Instantaneous COF of CFM; (**b**) instantaneous COF of PFM; (**c**) mean COFs of the CFM and PFM; (**d**) wear depths of the CFM and PFM.

Figure 5, Figure 6 and Figure 7 illustrate the test results of COFs and wear depths of CFM and PFM with regard to the applied load, rotating speed and ambient temperature. The COF of CFM is greater than that of PFM under the same working condition. This can be interpreted as follows. The COF is determined by the ratio of shear stress and normal load. Since the hardness of CFM is much larger than that of PFM, the shear stress generated by asperities contact in CFM is greater than that generated by asperities contact in PFM. Moreover, the COF variation range of CFM is relatively smaller than that of PFM under the same rotating speed and ambient temperature conditions, thus the CFM can withstand higher speed and temperature compared with PFM. However, under the applied load condition, the COFs of CFM and PFM have similar variation ranges. In conclusion, CFM does not simply have a higher torque transfer capacity than PFM but is less affected by the ambient temperature and rotating speed. Moreover, the wear amount of PFM is less than that of CFM under the various working conditions. This phenomenon can only suggest that the PFM is more likely to form a smooth friction surface, thus reducing the wear amount. It should be noted that severe wear or smoothness indicate the failure of friction material. Additionally, in the process of testing, the CFM makes more noise than PFM. The noise produced by CFM is irregular and occasionally accompanied by screaming, while the noise of PFM is low and smooth. Therefore, CFM is more suitable for clutches or brakes that require a higher and more stable torque transfer capacity, whereas PFM is a better choice for urban vehicles or machines to reduce noise.

### 4.1. Applied Load

As shown in Figure 5a,b, the COF of CFM is far larger than that of PFM under the same applied load condition. Moreover, as shown in Figure 5c, the mean COF of CFM almost decreases linearly with the increases of applied load. The reason has been mentioned above that the increase of applied load brings about the decrease in the ratio of shear stress and normal load. Additionally, under the dry condition, the increase of applied load can enlarge the asperity contact area which is identified as a smoother surface. Accordingly, as the applied load increases, the fluctuation of instantaneous COF becomes more and more gentle. However, when the applied load is less than 80 N, the mean COF of PFM increases gradually; when the applied load exceeds 80 N, the mean COF of PFM begins to decline slowly. It should be noted that the elastic modulus of PFM is much smaller than that of CFM. Since the real contact area increases with the increasing applied pressure, the contact area between the fibers and pin also increases leading to a slight increase of COF. As the applied pressure continues to increase, the resin mainly adhered to the protuberance area of the fibers will withstand most of the applied load, and the surface of PFM becomes glossy. The shear stress generated by resin is smaller compared with binders, so the COF decreases.

As illustrated in Figure 5d, the wear depths of CFM and PFM present an opposite trend with the increase of applied load. To be more specific, the wear depth of CFM increases gradually with the increasing applied load, but its growth rate decreases progressively. Based on the Archard Wear Law, the applied load is proportional to the wear amount of CFM [27]. However, the asperities in CFM are prone to elastic deformation under high applied load, which indicates that the number of asperities decreases significantly. Thus, the growth rate of wear depth decreases with the increase of applied load. Moreover, the wear depth of PFM is inverse to the applied load. As the applied load increases from 20 N to 120 N, the wear depth falls from 0.026 mm to 0.001 mm. With the increase of applied load, the contact area increases and the binders gradually withstand the applied load. Therefore, the contact surface becomes smoother and smoother, leading to a gradual reduction in wear amount.

### 4.2. Rotating Speed

As illustrated in Figure 6a, the variation of rotating speed can only cause slight fluctuations in the instantaneous COF of CFM; moreover, the maximum fluctuation of the mean COF of CFM is only 0.02 as presented in Figure 6c. Thus, the rotating speed can hardly affect the COF of CFM under the dry condition. However, the rotating speed has a dramatic influence on the mean COF of PFM, which decreases conspicuously from 10 rpm to 1000 rpm, then increases slightly, and the variation range is 0.005–0.310. This phenomenon can be interpreted as follows: Since the rotating speed is proportional to the heat flux, the increasing rotating speed can contribute to the dramatic increase of surface temperature. However, the thermal conductivity of PFM is much lower than that of CFM. As the rotating speed increases, the surface temperature of PFM is much larger than that of CFM. Accordingly, the hardness of PFM decreases, leading to the decrease of the shear stress, then resulting in an exponential decrease of COF under the speed range of 10–1000 rpm. Subsequently, the resin in PFM is softened and discomposed under high rotating speed. The friction surface with low resin content is more porous and rough, leading to a slight increase of COF.

Figure 6d demonstrates the wear depths of CFM and PFM with regard to the rotating speed. As the rotating speed increases, the wear depth of CFM increases slightly at first, and then increases almost linearly. As the rotating speed increases from 10 rpm to 100 rpm, the wear depths are 0.005 mm and 0.008 mm, respectively. The reason is that the low rotating speed corresponds to the low surface temperature, and the disc is in the abrasive wear status contributing to the stable wear depth. After that, the wear depth increases linearly; since the rotating speed corresponds to the sliding distance, the test results of CFM also confirm the correctness of the Archard Wear Law [27]. As to the wear depth of PFM, it decreases gradually at first, followed by a slight increase. To be more accurate, as the rotating speed increases from 10 rpm to 500 rpm, the wear depth decreases from 0.012 mm to 0.010 mm, finally decreasing to the minimum value of 0.005 mm. The reason is that as the rotating speed (heat flux) increases, the hardness of PFM decreases significantly leading to the decrease of the number of asperities, thus resulting in the decrease of wear amount. Subsequently, the resin begins to discompose, and the fibers are exposed to the friction surface resulting in a slight increase of wear depth.

### 4.3. Ambient Temperature

As shown in Figure 7, the ambient temperature has a significant influence on the COFs of CFM and PFM. As demonstrated in Figure 7c, the mean COF of CFM firstly increases progressively within the temperature range from 25 °C to 100 °C, then it varies slightly, whereas it begins to decrease above 175 °C. When the surface temperature reaches 250 °C, the fluctuation of instantaneous COF becomes violent. As for the PFM, it is known that the resin in the friction surface is softened and discomposed easily as the ambient temperature increases, leading to the decrease of COF. Therefore, the COF of PFM decreases apparently with the increase of ambient temperature. However, as the resin decomposes, the fibers are exposed on the friction surface and withstand the applied load. Consequently, when the temperature is in the range of 175–325 °C, the COF remains stable. Subsequently, the fibers are polished gradually and the surface roughness also decreases, which leads to the continuous decrease of COF. As discussed above, under the temperature range of 25–400 °C, the COF variation range of PFM is far larger than that of CFM, which is 0.025–0.27. These phenomena all confirm that the influence of ambient temperature on the PFM is much stronger than that of CFM. 

Figure 7d demonstrates the wear depths of CFM and PFM with regard to the ambient temperature. When the temperature increases from 25 °C to 100 °C, the wear pattern of CFM is abrasive wear, thus the wear depth changes slightly. However, as the ambient temperature continues to increase, the wear depth increases dramatically. The reason is that the properties of CFM change with the increasing temperature, and the most concrete manifestation is the gradual decrease of the hardness of CFM. However, with the increase of ambient temperature, the wear depth of PFM varies in a cosine pattern. As the ambient temperature increases, both the COF and the shear stress produced by asperities contact decrease apparently, leading to the decline of wear depth. However, the resin decomposes with the increasing temperature, resulting in the embrittlement of fibers, and finally leading to the increase of wear depth. When the ambient temperature reaches 400 °C, the resin adheres to the friction surface in liquid form, thus contributing to the decline of wear depth.

### 4.4. Wear Evolution

**Figure 8:**
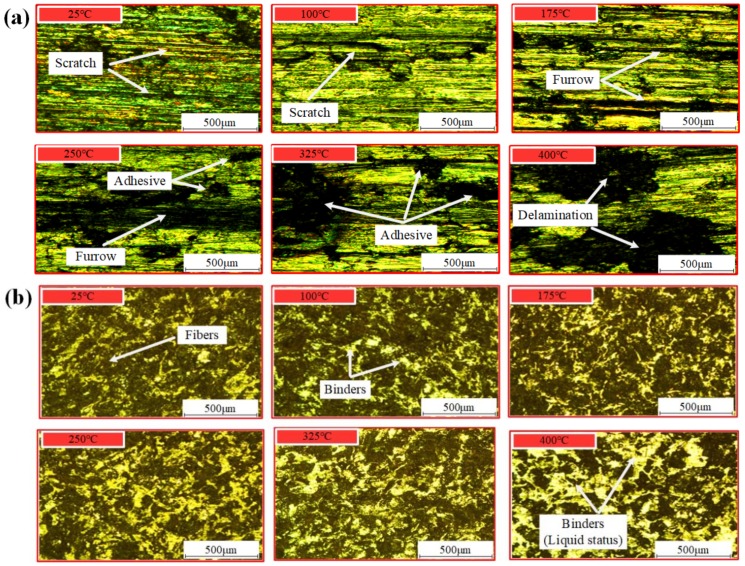
Metallographic micrograph of worn surface of the disc. (**a**) CFM; (**b**) PFM.

The characteristics of the worn surface after the test can reveal the wear mechanisms. Since the friction properties of friction materials are directly related to the temperature, the metallographic figures of CFM and PFM are presented under various ambient temperature conditions as shown in Figure 8, which can be used to analyze and evaluate their wear processes over the service life. It is known that the hardness of CFM falls down as the temperature increases. When the temperature is 25 °C, the hardness of CFM is high enough, and there are slight scratches and abrasive particles on the friction surface, which are identified as abrasive wear. When the temperature rises to 175 °C, furrows appear on the friction surface, and the wear pattern changes to furrow wear. As the surface temperature reaches 250 °C, the furrows are increasingly wider and deeper. When the temperature rises to 325 °C, the friction material begins to soften, thus the asperity contact begins to adhere. Under such high temperature, not only does the difference of the expansion coefficient between the metal and non-metal material of CFM occur, but also the shear stress caused by friction force leads to cracks perpendicular to the motion direction. After the temperature rises to 400 °C, CFM has been completely softened and the organization of CFM is destroyed. Accordingly, the CFM begins to peel off, which is recognized as peeling wear. 

As shown in Figure 8b, as the ambient temperature increases from 25 °C to 175 °C, the high temperature in the friction zone leads to the thermal decomposition of fibers in PFM, then resulting in the reduction of shear resistance. Thus, the contact area becomes smooth and the friction performance decreases. When the temperature continues to rise to 250 °C and 325 °C, the hardness of PFM gradually decreases, and the friction modifiers and most fibers begin thermal decomposition. Thus, the surface roughness and the shear stress increase. When the temperature reaches 400 °C, the thermal decay phenomenon deteriorates seriously. The resin and other binders spill over the friction surface, which can be considered as the lubricant. Therefore, the shear stress produced by asperities contact reduces rapidly, and the friction properties of PFM plummet to failure.

## 5. Conclusions

In order to evaluate and compare the friction and wear characteristics of the CFM and PFM during the clutch engagement process in the dry condition, the pin-on-disc tests are conducted with applied load, rotating speed and ambient temperature taken into account. The main conclusions are as follows:

1. Both the COF and wear depth of the CFM are much greater than these of PFM under the dry condition. The failure of CFM is caused by the variation of wear mechanism. With the gradual deterioration of the working condition, CFM will successively experience the abrasive wear, furrow wear, adhesive wear and peeling wear. Moreover, the failure of PFM is related to the wear of fibers and the softening and decomposition of resin, which will produce a much smoother surface.
2. As to CFM, the applied load can result in a linear decrease in COF, whereas the rotating speed can only cause a slight fluctuation in COF. Additionally, the ambient temperature can lead to a parabolic decrease in COF. The temperature range of 100–175 °C is most suitable for CFM, where the COF is high enough and the wear is slight.
3. As for PFM, with the increase of applied load, the COF remains stable at first, and then decreases slowly. The rotating speed and COF are inversely proportional. With the increase of ambient temperature, although the COF between 175–325 °C is relatively stable, the overall variation trend of COF is significantly decreased.
4. Under such three working conditions, the wear depth of CFM changes linearly. However, the wear depth of PFM varies greatly: The wear depth decreases inversely with the increase of applied pressure; with the increase of rotating speed, it decreases at first, and then increases slightly, and finally remains stable; it changes in cosine form as the ambient temperature increases.

## Figures and Tables

**Table 1:** Experimental conditions.

Factors	Applied Load (N)	Rotating Speed (rpm)	Surface Temperature (°C)
1	20, 40, 60, 80, 100, 120	500	175
2	60	10, 100, 500, 1000, 1500, 2000	175
3	60	500	25, 100, 175, 250, 325, 400

**Table 2:** Thermophysical properties of the pin and disc.

Factors	65Mn Steel	CFM	PFM
Poisson’s ratio	0.3	0.3	0.12
Elastic modulus (GPa)	160	6.2	0.27
Specific heat J/(Kg·°C)	487	460	1610
Density (Kg·m^3^)	7800	5500	1125
Thermal conductivity (W/m·°C)	45.9	9.3	0.241
